# Comparative Analysis of the Macroscale Structural Connectivity in the Macaque and Human Brain

**DOI:** 10.1371/journal.pcbi.1003529

**Published:** 2014-03-27

**Authors:** Alexandros Goulas, Matteo Bastiani, Gleb Bezgin, Harry B. M. Uylings, Alard Roebroeck, Peter Stiers

**Affiliations:** 1 Department of Neuropsychology and Psychopharmacology, Maastricht University, Maastricht, The Netherlands; 2 Department of Cognitive Neuroscience, Maastricht University, Maastricht, The Netherlands; 3 Institute of Neuroscience and Medicine – 4, Forschungszentrum Juelich GmbH, Juelich, Germany; 4 Rotman Research Institute of Baycrest Centre, University of Toronto, Toronto, Ontario, Canada; 5 Department of Anatomy and Neuroscience, VU University Medical Center, Graduate School Neurosciences Amsterdam, Amsterdam, The Netherlands; Hamburg University, Germany

## Abstract

The macaque brain serves as a model for the human brain, but its suitability is challenged by unique human features, including connectivity reconfigurations, which emerged during primate evolution. We perform a quantitative comparative analysis of the whole brain macroscale structural connectivity of the two species. Our findings suggest that the human and macaque brain as a whole are similarly wired. A region-wise analysis reveals many interspecies similarities of connectivity patterns, but also lack thereof, primarily involving cingulate regions. We unravel a common structural backbone in both species involving a highly overlapping set of regions. This structural backbone, important for mediating information across the brain, seems to constitute a feature of the primate brain persevering evolution. Our findings illustrate novel evolutionary aspects at the macroscale connectivity level and offer a quantitative translational bridge between macaque and human research.

## Author Summary

What are the commonalities and differences of human brains when compared to the brains of other primates? The brain can be conceived as a complex network. Its topological properties constrain its function. Ethical and technical reasons necessitate the use of animal brains, like the macaque monkey, as models for the human brain. However, evolutionary changes, including “brain rewiring”, might result in unique human features. Hence, a detailed and quantitative comparative analysis of the connectivity of the brains of the two species is needed. Here, we undertake this task by adopting techniques analogous to those used in comparative studies in other scientific fields. Our approach reveals converging but also diverging wiring patterns. The brain of the two species as a whole is similarly wired. The majority of the brain regions appear to have evolutionary conserved connectivity patterns while for certain regions this appears not to be the case. We also uncover an evolutionary conserved “structural backbone” in the brain of the two species. Our findings highlight common and unique “wiring properties” of the brains of these two primate species and offer a quantitative basis for translating findings from macaque research to human research.

## Introduction

Over a century of research has revealed that the brain is inhomogeneous and can be divided based on functional, macro- and micro- structural criteria [1]–[4]. The regions resulting from such a division are linked through fibre bundles that constitute the neural substrate for the exchange of information between the regions [5]. Early investigators highlighted the importance of the structural connections of a region to its functions, thus establishing the ground of structure-function dependencies and pinpointing the importance of brain connectivity for fundamental and clinical research [1], [6]. In recent years, studies offered evidence for the close relation of structural connectivity and function in the mammalian brain [7]–[9]. Hence, regions with similar connectivity might be involved in similar functions, and large scale connectivity constitutes a guide to cognition [10].

Due to ethical and methodological reasons our most detailed knowledge of the brain originates from animal research. Specifically, the macaque brain serves as a model for the human brain, but such extrapolations might be inaccurate due to rewiring and/or expansion during primate evolution [11]–[13] masking out unique features of the human brain [14]. This has important consequences for translating macaque research to humans, which is valuable for cognitive, systems and clinical neuroscience. Hence, there is the need for examining if classical homology criteria such as similarity of connectivity patterns [15], [16] are satisfied.

Diffusion weighted magnetic resonance imaging (dwMRI) is used for the examination of the structural connectivity of the brain in vivo and for comparing the structural connectivity of the human and macaque brain [17]–[19]. However, up to date studies focus on a small subset of brain regions, examining particular fasciculi or lack direct quantitative interspecies comparisons. Hence, interspecies similarities and discrepancies of connectivity patterns and topological features at the whole brain level remain largely concealed.

In addition, dwMRI is used for constructing in vivo the whole brain “wiring diagram” of humans, i.e. the human connectome [20]. Connectome analysis treats the brain as a complex network and employs tools from network science for unravelling key properties that are pivotal for its proper function and uncovering topological alterations related to mental disorders [21]–[24]. Recent work highlights key properties of the macroscale connectivity of the macaque brain [25] hinting at potential differences and similarities between the “connectome properties” of the two species, but with no explicit quantitative comparisons taking place.

To complement and surpass limitations of previous comparative studies, we perform a direct comparative quantitative analysis of the macroscale connectional architecture of the macaque and human brain. We adopt a macroscale parcellation scheme called the Regional Map (RM) [4], [26] and we construct whole brain species-specific connectomes, with the aid of dwMRI for the human and a neuroinformatics database for the macaque brain. Subsequently, we quantify the similarity of connectivity patterns, global topological features, and topology of the brain regions of the two species. This approach succeeds in uncovering preserved and divergent features of the macroscale connectional architecture of the brain of these two primate species.

## Materials and Methods

### Whole brain parcellation scheme

**Figure 1:**
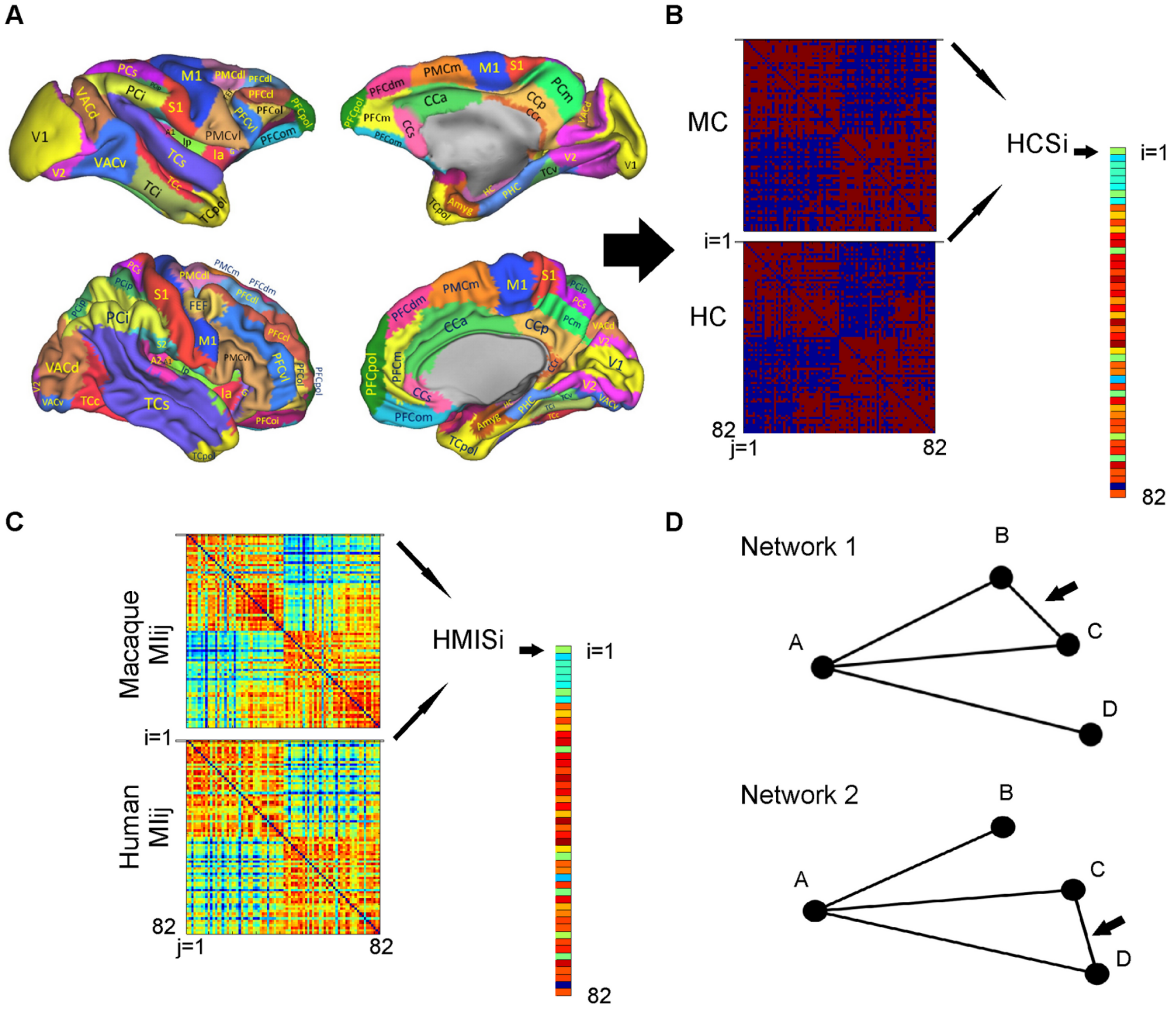
Schematic depiction of the main points of the analysis. A. The RM was used in order to construct the connectivity matrices of the two species, i.e. the MC and HC (panel B). The resulting connectivity matrices have i = 82 rows and j = 82 columns. B. Example of the calculation of the HCS for region i = 1. The entries of row 1 from the MC and row 1 from HC were used, resulting in entry HCS_i = 1_. The same procedure was used for all regions, resulting in a 1×82 vector HCS containing the HCS indexes of all the regions. C. Example of calculation of the HMIS for region i = 1. The MC and HC were used for the separate calculation of the matching index matrices MI_ij_ (one for each species separately). The HMIS index for region i = 1 is calculated from the entries of row 1 of the matching index matrices of the two species. The procedure is repeated for all regions, resulting in a 1×82 vector HMIS containing the HMIS indexes of all the regions. D. Toy networks demonstrating differences of the HCS and the HMIS. Given two hypothetical “brains” that form a network with 4 regions and 4 connections the HCS and HMIS indexes are calculated for brain region A. On the one hand, the HCS index is equal to 1 (perfect similarity), since region A in both networks is connected exactly to the same regions, i.e. nodes B, C and D. On the other hand, the HMIS index is −0.5, indicating a divergence of the connectivity similarity profiles of region A in networks/brains 1 and 2. These discrepancies arise from the “rewiring” of the connection marked with an arrow.

For the whole brain examination of both species we employed a map specifically designed for this purpose, the RM [4], [26] (Fig. 1 A). This map consists of putative homologues between the two species based on structural, macroscopic and functional criteria. Its level of coarseness is dictated from the size of regions that are discernible in both human and macaque brains [4]. No connectivity criteria were used for the delineation of the various regions constituting the RM. The RM was delineated on the F99 standard brain which is based on an MRI scan of one macaque brain. Subsequently, the RM was morphed to match the human brain by using macroscopic and functional landmarks [27], [28]. In total 82 regions (41 for each hemisphere) constituted the RM that we used (Table S1). We should note that the use of the RM is necessitated by the lack of an unequivocal “standard” microstructure based map for even the brain of one species, let alone a “standard” microstructure based comparative map [29], . Moreover, the regions constituting the RM are larger than regions defined based on e.g. cytoarchitecture, the so-called cortical fields, and one RM region might include various such cortical fields. This level of granularity of the RM was deliberately chosen in order to circumvent controversial issues with respect to macaque-human cortical field homologies across the whole brain, like the presence of more cortical fields in the human brain and/or duplication of certain cortical fields [4], [29], [30], [31].

### Macaque whole brain connectome

We used the RM and the CoCoMac database (http://cocomac.g-node.org) to assemble the whole brain connectome of the macaque. The database was accessed on December 2010. Briefly, the CoCoMac database consists of entries describing the presence of a structural connection between two regions, as revealed by tracing studies, and have the format: region A has an efferent connection with region B. The database contains over 400 studies spanning several decades and thus represents a current best estimate of the macroscale connectivity of the macaque brain. Different researchers use different maps with divergent nomenclature. In order to link the different maps the database contains relation codes with the format: Region A of map X is identical to region B of map Y. Dedicated algorithms and algebra is used to map regions of one map to regions of a “reference” map [32], [33]. In the current study, the RM functioned as the “reference” map and thus available connectivity information contained in the database was represented as an NxN connectivity matrix, where N = 82 the regions constituting the RM. A non-zero matrix entry A_ij_ denotes the presence of a connection from region i and j. In order to compare the connectivity of the macaque and human brain (see below), and since directionality of structural connections cannot be inferred in vivo in the human brain, the directed connectivity matrix of the macaque was symmetrized and binarized by taking into account all connections irrespective of their strength. The resulting macaque connectome (MC) consisted of 1857 undirected connections/edges between 82 regions/nodes. The binarization step is necessary since the connectomes of the two species were assembled from different modalities. DwMRI and tractography is not adequate for inferring density of connections [34] contrary to invasive tracing techniques in monkey studies. This limitation and the fact that certain network metrics employed for cross-species comparisons involve cross-matrix operations (see below), do not allow the use of a weighted approach, since the weights obtained from the different modalities are not comparable (see also Discussion).

### Human whole brain connectome

#### Data acquisition

Whole brain scans of five healthy subjects (2 females, age mean: 29.4 std: 3.2) were acquired after obtaining written informed consent. Data acquisition and preprocessing are described in [24]. Briefly, data were collected in a Siemens 3T MAGNETOM Allegra MR scanner equipped with a high slew-rate head gradient-coil (amplitude 40 mT/m, slew rate 400 T/m/s) and an 8-channel phased-array head RF-coil was used to acquire the data. A double refocused spin-echo diffusion sequence was used to acquire 131 volumes of data, with TR = 6600 ms, TE = 94 ms, b-value = 3000 s/mm^2^, 88×88 matrix, 52 axial slices, 2.5×2.5×2.5 mm^3^ voxels, partial Fourier = 6/8 and a bandwidth of 2840 Hz/pixel (echo-spacing 0.4 ms). A total of 120 diffusion gradient directions were acquired with 11 unweighted (b = 0 s/mm^2^) volumes acquired after every 12 gradient directions and including the first and last volumes. A T1-weighted 3D MPRAGE scan (TR = 2250, TE = 2.6 ms, flip angle = 9°, 256×256 matrix, 192 sagittal slices, 1×1×1 mm voxels) was acquired for gray/white matter boundary segmentation.

#### Voxel-wise diffusion model estimation

A multi-direction high angular resolution diffusion imaging based model has been used to estimate the voxel-wise orientation of neuronal fibers. Constrained spherical deconvolution fiber orientation distributions were reconstructed [35] over a five-fold tessellated icosahedron. This technique was selected for its robustness in estimating orientational distributions from high angular resolution diffusion imaging data. Moreover, fiber orientation distributions represent actual fiber orientation distributions rather than water-bound spin displacements, which leads to stable and accurate local orientations that are very beneficial for both local and global tractography purposes [36].

#### Tractography algorithm and parameters

A local probabilistic tractography algorithm was used in this study. The employed algorithm uses orientations sampled from the fiber orientation distributions at each step and initializes a great number of streamlines per seed point in a way similar to the PICo algorithm [37]. This tractography algorithm has shown good performance based on empirical data [24] and phantom based evaluations [38]. Per seed point, 3000 streamlines were initiated within a sphere whose center corresponded to the center of every white matter boundary voxel and whose radius has been set to half the voxel size (1.25 mm). The step size was set to 1 mm and the angular thresholds to 30°.

Fractional anisotropy maps were thresholded at a value of 0.1 in order to obtain the white matter waypoint masks. These are binary masks containing only those voxels where fibers are allowed to propagate. The tractography algorithm used in the present study was run in original diffusion data space. Therefore, we have chosen to use median filtered fractional anisotropy masks computed in that same space, instead of white matter masks obtained from T1-weighted volumes segmentation, in order to achieve maximum integrity and alignment of white matter masks to the diffusion data. To avoid influences on fractional anisotropy such as partial volume effects at the white/grey matter boundary and in those voxels where more than two diffusion directions are reconstructed a 3-dimensional median filter has been applied to the thresholded white matter volumes to fill holes in the masks. Furthermore, in the white matter binary masks, white matter boundary voxels were always included in the volume after having thresholded the fractional anisotropy mask and used the median filter. Fibers shorter than 10 mm or longer than 200 mm were removed. Moreover, looping fibers (i.e. fibers that return to already explored voxels) are excluded from the analysis. Probabilistic local multi-direction tractography was performed using the MRtrix package [35]. To move from a very high resolution tractography result that connects all ∼30000 voxels in the white matter bound voxel set to the weighted connectivity matrix based on the 82 regions of the RM, we employed the same connectivity index as defined in [39]. This index of connectivity between two regions is given by the sum of the weights connecting all the voxels between region A and region B and vice versa, normalized by the sum of the number of seeds used in each region. Thus, any non-zero weight connecting any voxel in one parcel to any voxel in another in either of the two directions connects the two parcels in the final symmetric adjacency matrix [39]. Such step aims at reconstructing the weighted 82×82 connectivity matrix eliminating the effect of patch-area normalization. To create the human connectome (HC) the symmetrized weighted matrices obtained from each individual were averaged. Subsequently, a certain threshold has to be applied to the probabilistic tractography results. Thresholding constitutes a necessary step in connectome reconstruction [21], [22]. No thresholding option provides a definite answer about the “true” underlying connections for the whole brain. Consequently, the threshold was chosen in a way not to uncover the “true” connections, but to render the MC and HC comparable, which serves the purpose of the current study. Hence, only the highest weights were selected and their number was chosen to match the number of connections of the MC. Finally, the HC was binarized. Hence, the MC and HC are binary undirected matrices with the same number of nodes and connections, corresponding to a density of 0.559. It should be noted that the vast majority of the connections (85%) in the average matrix were present in at least 4/5 of the individual matrices (after thresholding at the same density). Therefore, averaging across the participants did not bias towards connections that have particularly high weight in a minority of the subjects. A very good interindividual similarity was also observed with a high correlation between the unthresholded weighted matrices of each subject (mean: 0.86 range: 0.83–0.90).

### Comparing the macaque and human connectome

We aimed at examining key topological properties of the MC and HC. Below we introduce the various network metrics, defined at a region-wise or whole brain level, with relevant references.

Given two matrices A and B, representing the MC and HC respectively, we computed the intersection network X defined as:
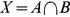
(1)The number of edges L_x_ of the intersection network denotes the common edges/connections of the MC and HC. L_x_ divided by the total number of edges L( = 1857) in each network A and B offers a measure of similarity of the two networks. Hence, the ratio L_x_/L ranges from 0 to 1, indicating no overlap and complete overlap respectively of the edges of networks A and B.

We next procedeed to a region-wise analysis and sought to quantify the overlap of the connections of the assumed homologues of the MC and HC. This was performed with the Homologue Connectivity Similarity (HCS) metric:
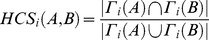
(2)The interspecies overlap/intersection of connections of region i in the macaque and human brain, represented by A and B respectively, is denoted by 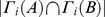 and the union with 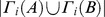 with 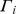 denoting the set of neighbours of node i and 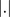 the size of the set (see Fig. 1 B). Hence, for each region i = 1…82 of the RM the HCS_i_ ranges from 0 to 1 and indicates respectively low and high interspecies connectivity similarity of the assumed homologue region i.

Subsequently, we quantified a “second-order” similarity of homologous regions, i.e. their connectivity similarity profile with the rest of the brain. To this end, we used the matching index [40] for A, denoting MC (the same for B denoting the HC):
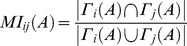
(3)


This resulted in one MI matrix for each species, with the row i capturing the connectivity similarity of region i with all the other brain regions of the same species. Hence, each row of each MI matrix can be conceived as a “connectivity similarity profile” of a region constructed for each species separately. In order to quantify if the connectivity similarity profile of putative homologous regions was preserved, we calculated the correlation between row i = 1…82 of the MI matrices (without including the diagonal entries of MI_A_, MI_B_,, i.e no self-similarity values). This resulted in the Homologue Matching Index Similarity (HMIS):
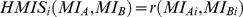
(4)with r denoting Pearson's correlation coefficient of row i of the two MI matrices (Fig. 1 C).

Main aspects of the topology of the RM regions in the whole brain network, i.e. centrality and clustering, quantifying “importance” and “segregation” of regions [41], were examined by calculating the betweenness centrality (BC) [42], eigenvector centrality (EC) [43] and clustering coefficient (C) [44]. Segregation in this context implies a tightly interconnected neighborhood of a brain region, allowing “cross-talk” and exchange of information, while “importance” refers to highly central regions that are topologically ideal for information integration [45]. All these metrics were calculated for each species separately by using the formulas for binary undirected networks described in [45], [46]. In order to assess perseverance of the centrality and clustering of the brain of the two species, Spearman's rank correlation between the same network metrics from the two species was computed. A statistically significant (positive) correlation would indicate the evolutionary perseverance of each network metric.

Lastly we examined the presence of a rich club organization, indicating the presence of a “structural backbone”, quantified with the rich club coefficient (RCC) [47]:
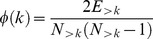
(5)where E_>k_ denotes the number of connections/edges that exist among nodes/regions that exhibit more connections than a given number k and N_>k_ denotes the number of nodes/regions that have degree higher than k, i.e. exhibit more connections than a given number k. The RCC is calculated for a range of k for a given network and for a number of randomized matched networks in order to estimate the RCC values expected by chance. This results in a normalized RCC:
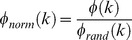
(6)Values higher than 1 for a range of values k indicate that the network is characterized by a rich club structure, with nodes with degree higher than k linked with more connections than expected by chance [47].

For each analysis, 10000 random networks, unless otherwise stated, matching each MC and HC in number of nodes, edges and degree distribution were created, with the use of a degree preserving algorithm [48]. The random networks were used for calculating p-values and z-scores of the network metrics. Thus, the random networks are used for obtaining “null” values for the metrics used. For the region-wise analysis, introducing multiple tests, we used a conservative Bonferroni correction.

For verifying the robustness of the findings of the topology of the original MC and HC, we performed the following control analyses. First, we perturbed the MC and HC by rewiring the network with a low probability, i.e. 0.1, while keeping the number of edges, nodes and degree distribution intact. That is, a pair of edges was swapped with 0.1 probability, thus introducing modest alterations to the network. Secondly, connections in empty positions were randomly and uniformly inserted. We inserted 10% of the initial number of connections, i.e. 1857*10% = 186 connections. Random networks for addressing the significance of the results in these “randomly enriched” MC and HC matched the new higher density. This type of analysis simulates in a simple way a scenario were previously absent connections appear to be present [49]. Techniques like the aforementioned ones were used for the examination of the robustness of features of the HC [21]. Additionally, we examined the effect of the choice of a particular parcellation by performing all the analyses on the connectomes in the exact same way, but assembled based on a different parcellation scheme [3]. Lastly, due to limitations of dwMRI-based local probabilistic tractography techniques in revealing long controlateral connections [24], [38] and the lack of complete information on such connections in the CoCoMac database, we performed a within hemisphere analysis for the left and right hemisphere seperately. To this end, we constructed the MC and HC as previously described but for each hemisphere seperately. The hemisphere-wise MC appeared very dense (left hemisphere:0.791 right hemisphere:0.792 density, compared to 0.559 for the whole brain connectome) and the HC was thresholded accordingly. The very high density of the hemisphere-wise connectomes poses a problem for a binary analysis. This is due to the fact that many topological properties of the original MC and HC will not differ from their rewired counterparts because of inefficient “space” for rewiring. Taking an illustrative case for example, the rich club analysis will reveal dense connections between regions at increased levels of k but such strong interconnectivity can be merely explained by the very high density of the network and is not therefore not “surprising”/statistically significant (for a similar discussion but a different direction see [50]). Therefore, we decided to adopt weighted networks and suitable weighted versions of the aforementioned metrics. As previously explained, the weights from the MC and HC are not comparable. Therefore, we restricted the weighted hemisphere-wise analysis to metrics that do not involve cross-matrix operations (thus the intersection and HCS were not computed). The weighted version of the HMIS involved equation (4) but operating on the rows of a “weighted matching index” matrix obtained as the cosine similarity between rows i and j of matrix A, denoting MC (the same for B denoting the HC):
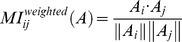
(7)The weighted version of EC, BC and C was computed as described in [45]. The weighted rich club can be formulated as follows [51]:
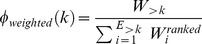
(8)with W_>k_ denoting the sum of the weights of the edges connecting nodes with degree higher than k and 
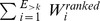
 denoting the sum of the E_>k_ first ranked (in decreasing order) edge weights in the whole network. For each analysis, 10000 random networks (corresponding to the “link and weight reshuffle” model in [51]) matching each hemisphere-wise MC and HC in number of nodes, edges and degree distribution were created, with the use of a degree preserving algorithm [48].

All network analysis was performed with the use of functions from the Brain Connectivity Toolbox (https://sites.google.com/site/bctnet/) [45] and custom scripts written in Matlab (Mathworks). The MATLAB code for the computation of the HCS is provided (Software S1). Brain renderings were performed with the following freely available software: Caret (http://brainvis.wustl.edu/wiki/index.php/Caret:About) and BrainGL (http://code.google.com/p/braingl/). For certain visualizations of the connections of the MC and HC a mean-shift edge bundling algorithm was used [52].

## Results

**Figure 2:**
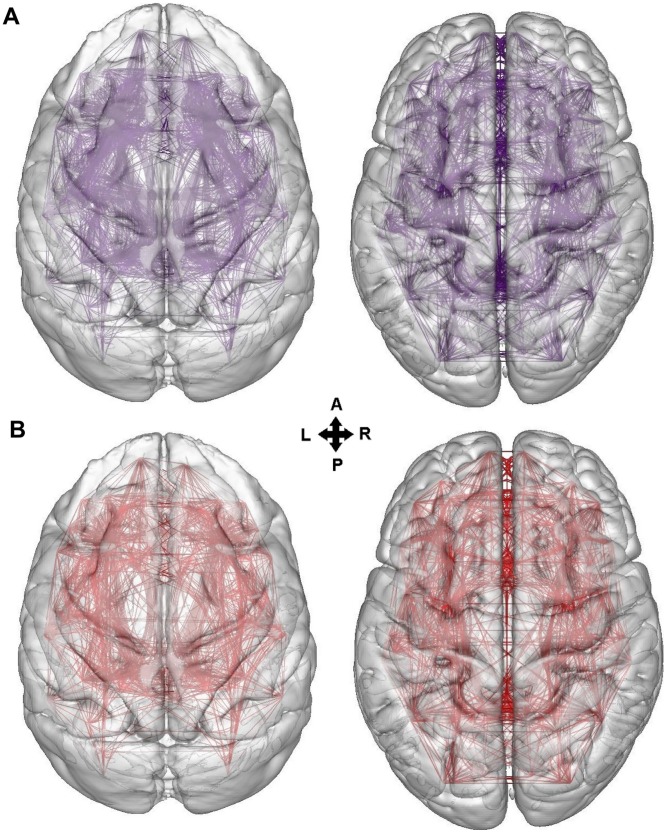
Overlap of the MC and HC. A. Rendering of the MC and HC. B. Rendering of the overlap between the MC and HC. The two connectomes exhibit a statistically significant overlap (see Results). The renderings were performed with BrainGL and a mean-shift edge bundling algorithm [52].

Fig. 2 depicts the MC and HC and their intersection. Their intersection, i.e. the L_x_/L ratio of the MC and HC was significant (L_x_/L_original_ = 0.754 p<0.001, L_x_/L_null_ mean = 0.599 std = 0.006, null values from 1000 random networks). Thus, the wiring of the macaque and human brain as a whole is more similar than expected by chance.

**Figure 3:**
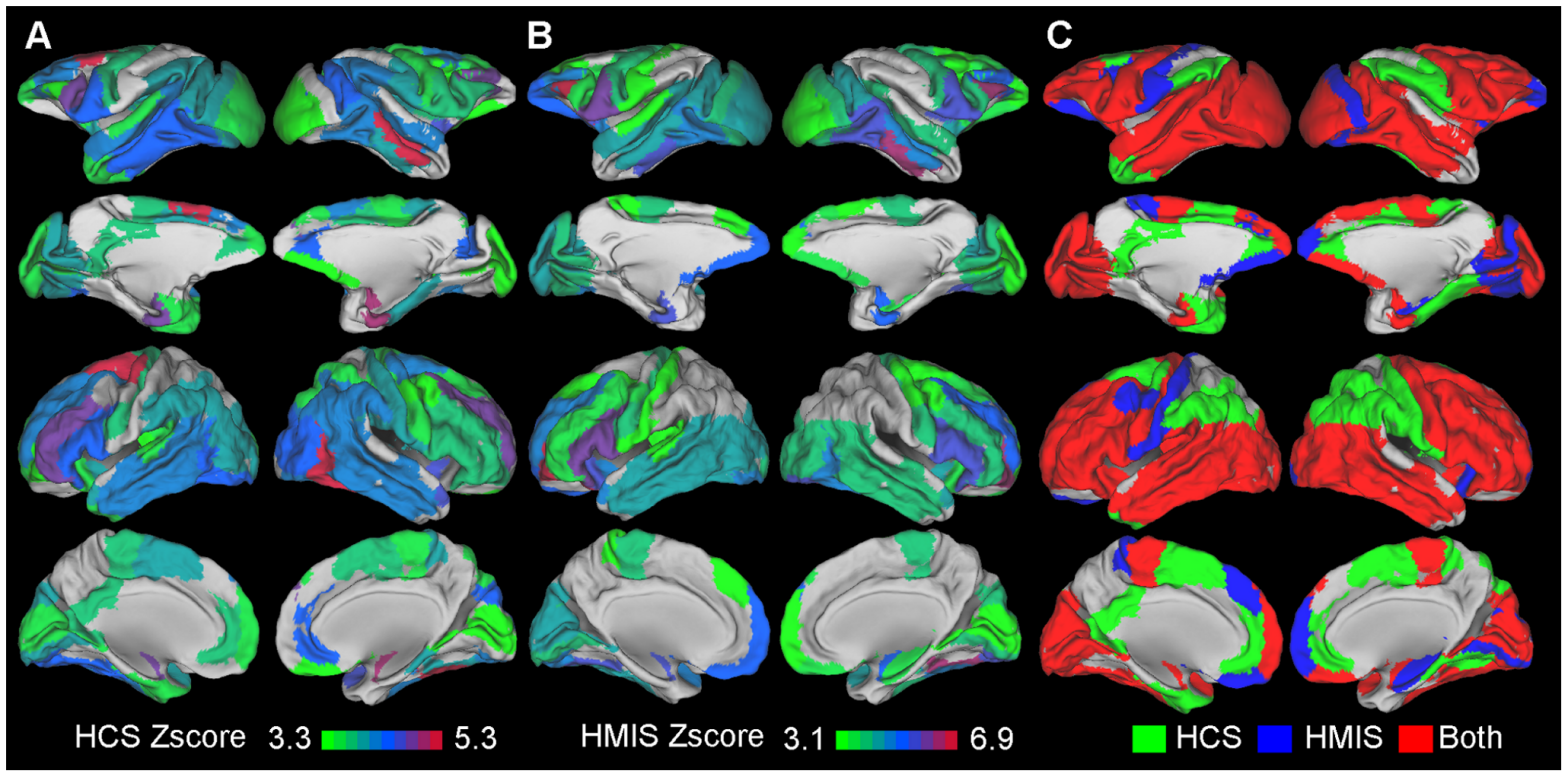
Renderings of the macaque and human regions exhibiting a significant HCS and HMIS index. A. HCS index B. HMIS index. In both panels only regions reaching significance are depicted (p<0.05 Bonferroni corrected). Colour coding denotes their corresponding z-score. C. Summary of results by colour coding the regions based on the preservation of both HMIS and HCS (red), only HCS (green), only HMIS (blue).

The region-wise analysis of the HCS revealed significant connectivity preservation for many RM regions. Specifically, a set of frontal, occipital and temporal regions exhibited significant preservation of their whole brain connectivity across the species. Mainly parietal and cingulate regions appeared to lack such preservation (Fig. 3 A, Table 1). In sum, 51 out of 82 RM regions exhibited significant HCS and therefore communicate with a significantly overlapping set of brain regions in both species.

**Table 1:** HCS and HMIS results for the RM regions for whole brain binary network analysis.

RM acronyms	Right				Left			
	HCS		HMIS		HCS		HMIS	
	z-score	p-value	z-score	p-value	z-score	p-value	z-score	p-value
PFCoi	3.08	0.0015	2.94	0.0014	3.31	0.0007	2.61	0.0042
PFCom	**3.39**	**0.0003**	**3.57**	**0.0001**	2.41	0.0135	**5.08**	**0.0001**
PFCol	**3.77**	**0.0002**	**6.29**	**0.0001**	**3.49**	**0.0006**	**6.85**	**0.0001**
G	2.62	0.0046	**3.77**	**0.0001**	**3.65**	**0.0002**	**5.45**	**0.0001**
PFCpol	2.90	0.0040	**3.30**	**0.0006**	**3.62**	**0.0002**	**5.15**	**0.0001**
PFCvl	**3.91**	**0.0001**	**4.19**	**0.0001**	**4.73**	**0.0001**	**3.79**	**0.0001**
PFCm	**4.35**	**0.0001**	3.11	0.0007	**3.73**	**0.0001**	2.10	0.0170
PFCcl	**4.86**	**0.0001**	**5.09**	**0.0001**	**4.28**	**0.0001**	**4.85**	**0.0001**
M1	**3.67**	**0.0004**	**3.86**	**0.0001**	**3.84**	**0.0003**	**3.91**	**0.0001**
PFCdm	2.04	0.0360	1.07	0.1390	2.12	0.0310	**3.36**	**0.0001**
FEF	**3.37**	**0.0006**	**3.43**	**0.0001**	2.59	0.0071	**3.75**	**0.0001**
PFCdl	**3.72**	**0.0003**	**3.82**	**0.0001**	**4.26**	**0.0001**	**3.14**	**0.0004**
PMCvl	**3.82**	**0.0001**	**5.62**	**0.0001**	**4.35**	**0.0001**	**5.76**	**0.0001**
PMCm	**3.80**	**0.0003**	2.57	0.0040	**4.04**	**0.0001**	2.64	0.0036
PMCdl	**4.26**	**0.0001**	**3.42**	**0.0001**	**5.30**	**0.0001**	3.16	0.0007
Tcpol	3.18	0.0012	2.47	0.0066	**3.66**	**0.0001**	2.11	0.0166
TCs	**4.29**	**0.0001**	**4.13**	**0.0001**	**4.26**	**0.0001**	**4.37**	**0.0001**
TCc	**5.13**	**0.0001**	**4.68**	**0.0001**	**4.45**	**0.0001**	**4.51**	**0.0001**
TCi	**4.01**	**0.0001**	**6.14**	**0.0001**	**4.41**	**0.0001**	**5.61**	**0.0001**
TCv	**3.33**	**0.0005**	1.94	0.0242	3.12	0.0010	1.87	0.0291
A1	3.07	0.0015	2.58	0.0036	**3.53**	**0.0006**	**3.26**	**0.0002**
A2	2.89	0.0039	2.16	0.0137	**3.66**	**0.0003**	**3.34**	**0.0001**
S1	**3.94**	**0.0001**	2.57	0.0046	3.33	0.0009	**3.27**	**0.0004**
S2	**4.11**	**0.0001**	3.20	0.0007	**4.05**	**0.0001**	**3.07**	**0.0006**
PCi	**4.21**	**0.0001**	0.01	0.5032	**4.03**	**0.0001**	0.48	0.3214
PCm	2.63	0.0089	2.17	0.0136	2.91	0.0058	2.49	0.0052
PCip	**3.67**	**0.0003**	0.54	0.2936	3.24	0.0019	0.87	0.1910
PCs	1.96	0.0441	1.90	0.0284	1.54	0.1036	1.94	0.0253
V1	**3.52**	**0.0001**	**3.39**	**0.0003**	**3.55**	**0.0002**	**4.48**	**0.0001**
V2	3.47	0.0007	**4.10**	**0.0001**	**4.09**	**0.0001**	**4.03**	**0.0001**
VACv	**4.27**	**0.0001**	**5.67**	**0.0001**	**4.32**	**0.0001**	**4.92**	**0.0001**
VACd	**4.32**	**0.0001**	**4.22**	**0.0001**	**3.94**	**0.0001**	**4.44**	**0.0001**
Amyg	**4.92**	**0.0001**	**4.99**	**0.0001**	**4.86**	**0.0001**	**5.60**	**0.0001**
PHC	**3.96**	**0.0001**	1.80	0.0337	2.74	0.0058	1.70	0.0432
HC	2.92	0.0025	**3.64**	**0.0002**	2.73	0.0033	3.11	0.0009
CCs	2.38	0.0160	0.65	0.2650	2.53	0.0102	0.05	0.4836
CCr	2.14	0.0239	0.12	0.4633	2.43	0.0122	−0.22	0.5884
CCp	3.26	0.0009	1.46	0.0727	**3.90**	**0.0001**	2.09	0.0164
CCa	2.27	0.0271	−0.79	0.7836	1.84	0.0646	−0.33	0.6341
Ia	**4.65**	**0.0001**	**4.47**	**0.0001**	**4.01**	**0.0001**	**4.78**	**0.0001**
Ip	1.34	0.1502	0.21	0.4200	1.18	0.1851	−0.03	0.5145

Regions exhibiting significant HCS and HMIS values (p<0.05 Bonferroni corrected) are in bold. The p-values and z-scores of the aforementioned metrics are also depicted.

The region-wise analysis of the HMIS revealed that 45 out of 82 RM regions, mainly involving frontal, temporal, occipital regions reached significance. Cingulate and parietal regions failed to reach significance (Fig. 3 B, Table 1). Hence, regions reaching significance seem to form the same “connectivity coalitions”, i.e. exhibit a statistically significant connectivity similarity profile with the rest of the brain regions in macaques and humans. This in turn can entail that their “functional coalitions” might also be preserved. Conversely, certain regions fail to reach significance and might suggest that “evolutionary rewiring” occurred in such a way that they formed distinct “connectivity coalitions” with the rest of the brain regions in the two species.

The HCS and HMIS results involve distinct but also overlapping sets of regions (Fig. 3 C, Table 1). Thus, they illustrate converging but also diverging aspects of distinct connectional characteristics of the brain regions of the two species.

The hemisphere-wise weighted HMIS analysis revealed broadly the same pattern with a notable difference: a subset of prefrontal and temporal regions did not reach significance (Table S2). Notably, even in this type of analysis cingulate regions failed to reach significance.

**Figure 4:**
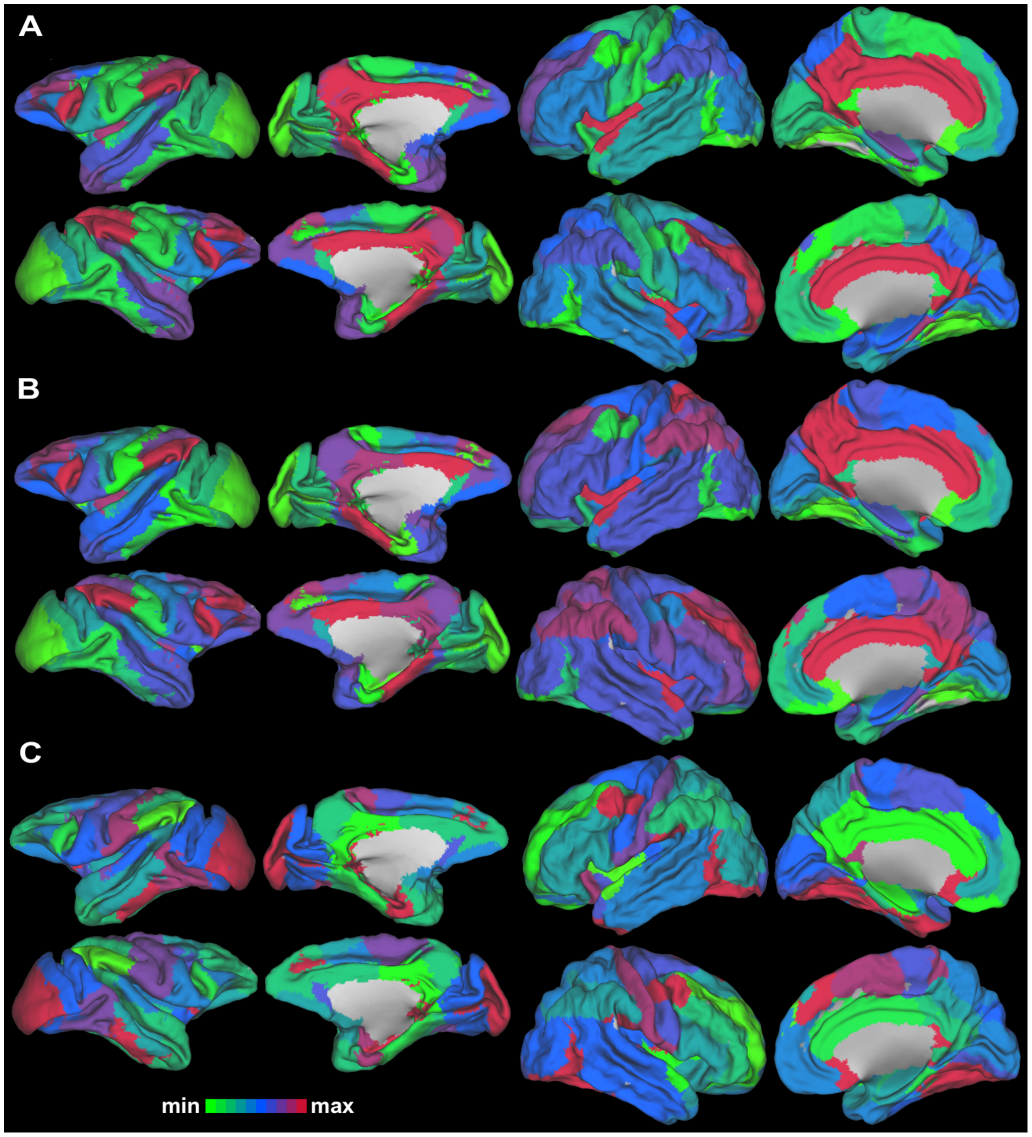
Centrality and clustering in the MC and HC. A. BC B. EC and C. C values for the regions of the two species. Despite some common patterns, e.g. high centrality of cingulate cortex regions, these network metrics do not significantly correlate across the species (see Results).

Fig. 4 depicts the results of the centrality and clustering analysis of the MC and HC. In both species, a general pattern is discernible with regions in “association” cortex exhibiting the highest centrality and regions in “primary” cortex exhibiting the lowest (Fig. 4 A B). Moreover, in both species the cingulate cortex appears as highly central (Fig. 4 A B). Additionally, little overlap was observed between the macaque and the human brain when taking into account the regions that are highly central (centrality>mean+1 std of the centrality of the RM regions) (Table S3). It should be noted however, that bilateral posterior cingulate cortex (CCp) and left inferior parietal cortex (PCi) exhibited high (>the mean+1 std threshold) BC and EC in both species indicating the perseverance of the prominent central role of these regions. However, a region wise correlation of the BC and EC values across the species revealed a relative high but not significant correlation (rho = 0.51, 0.52 respectively p>0.1). This might suggest that, at a whole brain level, there is a lack of perseverance of the topological importance of the assumed homologues of the macaque and human brain (see also Fig. S1 A B for scatterplots of the BC and EC values from the two species).

The C values for both species exhibited a “reversed” pattern with the centrality values: regions in “association” cortex exhibiting the lowest values and regions in “primary” cortex exhibiting the highest (Fig. 3 C). The C values across the species did not reach significance either (rho = 0.46 p>0.1). Therefore, the regions of the brains of the two species seem to exhibit different levels of segregation (see also Fig. S1 C for scatterplots of the C values from the two species).

The hemisphere-wise weighted anlysis of EC, BC and C led to a comparable picture (Table S4) with no significant correlation between these metrics in the two species.

**Figure 5:**
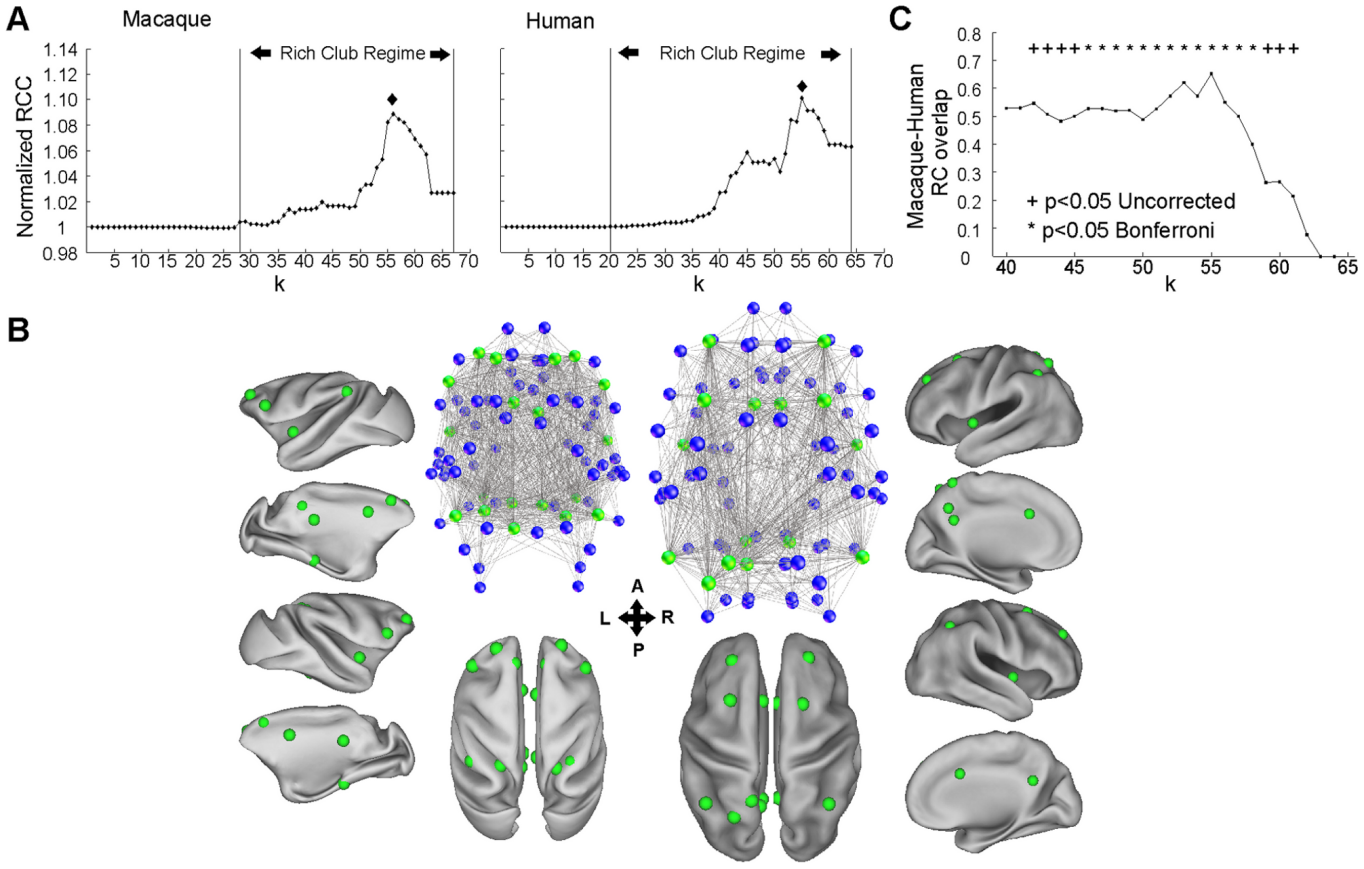
Rich club structure in the macaque and human brain. A. The normalized RCC suggests the presence of a rich club organization in both the human and macaque brains. For the unormalized curves see Fig. S3. The RCC obtained within the rich club regime (>1) were significantly higher than the ones obtained from random networks matched for node, edge and degree distribution (p<0.0001). B. Network and anatomical representation of the regions corresponding to the peak of the normalized RCC which is marked with a diamond in panel A (k = 56 for macaque and k = 55 for human). In the network representation, green and blue nodes represent rich club and non-rich club regions at level k respectively. Only connections involving at least one rich club region are depicted. The anatomical representation depicts the regions constituting the rich club on the inflated fiducials of both hemispheres of the brains of the two species. The spheres represent the centre of mass of the regions. Note the convergence of the rich club analysis to a highly overlapping set of regions in the two species. C. The observed overlap is significant within a range of the rich club regime. The significance was established by drawing randomly from a uniform distribution a number of regions from the MC and HC equal to the number of regions corresponding at each level k for the MC and HC separately. The procedure was repeated 10000 times and the overlap of these randomly drawn regions was computed forming a null distribution with which the original overlap values were compared.

A significant RCC highlights the presence of a rich club organization in both the MC and HC (Fig. 5 A, see also Fig. S3) in line with previous studies [22], [53]. Importantly, our direct comparative analysis that employed a parcellation scheme applicable to both species demonstrates that the regions forming a rich club exhibit a high and significant overlap (14/20), involving regions located at the frontal, parietal, cingulate and insular cortex (Fig. 5 B C, Table S5). This overlap is observed for a wide range within the rich club regime (Fig. 5 C). This indicates that not only the macaque and human brain exhibits a rich club organization, but that this structure constitutes an evolutionarily preserved structural backbone involving a highly overlapping set of regions in both species.

Since the regions constituting a rich club have a high degree, i.e. number of connections, and the degree is positively related to BC and EC [46], it is expected that the rich club regions will have higher BC and EC values when compared to non-rich club regions. We directly tested this prediction and found that rich club regions in both the MC (defining rich club and non-rich club regions by talking into account level k = 56 as a cutoff) and HC (defining rich club and non-rich club regions by talking into account level k = 55 as a cutoff) exhibit significantly higher BC and EC values when compared to non-rich club regions (p<0.001, permutation tests). Moreover, comparing the C values of rich club and non-rich club regions revealed the reversed relation: the rich club regions exhibited significantly lower C values when compared to non-rich club regions (p<0.001, permutation tests). Hence, the regions of the “structural backbone” in both the MC and HC when compared with the rest of brain regions, appears highly central, further underlying their topological importance in mediating information across the brain. Moreover, they appear less segregated, indicating limited connections, and hence possible anatomical paths for “cross-talk”, between the regions that they connect to.

Application of the weighted RCC to the left and right MC and HC seperately, led to similar results (Table S6 S7). One notable exception was the failure of the weighted rich club analysis to reveal a statistically significant rich club in the left HC. Hemispheric differences in network metrics have been reported [39] and this finding could signify a less prominent rich club structure in the left HC. However, given the high density of the network, and consequently a rather low cutoff used for considering connections in the HC to be taken into account, we suggest that this finding is the consequence of an inflated false positive rate obscuring the topology of the left HC.

For the whole brain analysis involving binary networks, control analysis gave rise to the following picture: The L_x_/L ratio on perturbed networks revealed that despite a slight decrease, as expected since scrambling of the networks was introduced, from the L_x_/L ratio calculated between the original MC and HC, the L_x_/L ratio remained significantly higher when compared to values obtained from random networks (L_x_/L_perturbed_ mean = 0.658 std = 0.003, L_x_/L_null_ mean = 0.599 std = 0.006, p<0.001 null values from 1000 random networks). The HCS, HMIS, and RCC analysis from networks derived from the perturbation analysis revealed that the results are robust (Fig. S2, S3). This also held true for the control analysis of random insertion of connections. The usage of a different parcellation scheme [3] led to significant and converging results as the ones obtained for the RM (L_x_/L_original_ = 0.712 p<0.001, L_x_/L_null_ mean = 0.558 std = 0.011 null values from 1000 random networks, see also Table S8, S9). The choice of a different parcellation scheme gave rise to significant and comparable results, albeit with less regions reaching significance (Table S8), something that might be attributable to the fact that this map was not “designed” to be applicable in both species. Hence, the above results conjointly underscore the robustness and relative independence of the results from parcellation scheme choices.

## Discussion

### Prefrontal, parietal and cingulate regions

Cortical expansion of the human cortex in relation to the macaque is more prominent in prefrontal, parietal and cingulate regions [54]. Our results suggest different degrees of perseverance of the macroscale connectivity of these regions during primate evolution.

An early view on the prefrontal cortex (PFC) suggests that it has been expanded in the lineage leading to humans [1], [55]. Expansion of the human PFC relative to the macaque PFC is supported by contemporary investigations [13] and is linked to unique human cognitive processes [56]. Moreover, PFC connectivity changes have also been proposed to underlie unique cognitive processes in humans [11]. A recent review [57] as well as functional [58] and structural [19] connectivity studies suggest comparable connections of the PFC of the two species. Additionally, quantitative analysis has revealed similar connectivity of macaque and human PFC regions with a small set of cortical regions (17). However, pronounced changes are reported for the arcuate and inferior fronto-occipital fasciculi of humans and macaques [19], [59]. Our study suggests a statistically significant preservation of distinct aspects of the wiring of several PFC regions across the species (Fig. 3, Table S2). Hence, unique features of the humans, i.e. “higher order cognitive processes/intelligence” attributed to the PFC, might not entail extensive reconfigurations of PFC connectivity in humans when compared to macaques.

The parietal cortex in macaques and humans consists of distinct subregions that are discernible on functional, connectional, macro- and microstructural criteria [3], [4], [60]. Comparative studies reveal similarities but also some differences of the functional and connectional architecture of the parietal cortex subregions [60]–[62]. Our whole brain quantitative analyses offer complementary evidence by revealing that certain lateral parietal regions reach a statistically significant connectivity pattern similarity, while the medial parietal ones do not (Fig. 3, Table 1 S2). This could entail a functional similarity of lateral parietal regions between the two species and a divergence with respect to the medial ones.

The anterior cingulate cortex (CCa) exhibits extensive connections with parietal, motor, frontal, insular and limbic regions. Such connectivity renders it suitable for bridging the motivational, cognitive and motor domains [63]. Functional evidence in humans and macaques pinpoint such an integrative role and involvement in decision making [64], [65]. CCa is highly central and part of the rich club (Fig. 4 5, Table S3 S4 S5 S7) a topological structural feature that might allow the involvement of this region in the aforementioned functional roles. Despite that CCa is part of the rich club in both species our results suggest a lack of preservation of its connectivity patterns (Fig. 3, Table 1, Table S2). This in turn might entail, alongside with potential preservance of certain functional properties, divergent functional roles of this region in the two species.

The posterior cingulate cortex (CCp) is a major node of the default mode network in both species, also involved in processes such as social cognition [66]–[68]. In addition, recent evidence from a functional study in humans suggests that this region exhibits dynamic properties subserving the integration of information from regions of distinct large scale networks [69]. The fact that CCp is central and part of the rich club in both species (Fig. 4 5, Table S3 S4 S5 S7) might constitute the structural basis for such integrative property reflected in functional measurements. Consequently, we hypothesize that such property will also hold for the macaque. However, the CCp appears to have not retained its connectivity with the rest of the brain (Fig. 3, Table 1 S2). Multimodal imaging of the macaque and human brain might be used to directly address if the aforementioned integrative functional property involving the CCp are common in the two species or a unique property of the human brain. Moreover, a possible rewiring of the CCp might have resulted in the reconfiguration of the neural circuitry, which seems also present in the macaque brain [68], underlying aspects of social cognition in humans.

### Centrality and clustering

A general pattern was discernable in both species: high BC and EC values were observed in “association” cortices and low ones in “primary” cortices. The C values exhibited the reverse pattern (Fig. 4, Table S4). However, none of these network metrics appeared to persevere primate evolution, suggesting different levels of centrality and clustering at the whole brain level (see also Fig. S1). Neverthless, regions in the cingulate cortices appeared highly central in both species (Fig. 4 A B, Table S4) in line with previous findings [24], [53]. Hence, cingulate cortex regions, despite the evidence for a different “wiring” in the two species (Fig. 3, Table S2), seem to have mantained their topological centrality, relevant for information integration.

### Rich clubs: A common structural backbone in the macaque and human brain

Our analysis demonstrates the presence of a rich club organization in both the MC and HC (Fig. 5, Table S5, S6, S7, S9) confirming and extending previous findings [22], [53]. Importantly, our comparative approach allowed us to demonstrate that the regions forming a rich club are highly converging with a significant overlap within the rich club regime (Fig. 5 B C, Table S5 S7 S9). Thus, this structural backbone is not only present in both macaques and humans, but also persevered through primate evolution, involving a highly overlapping set of regions in the two species. It is noteworthy, that the hemisphere-wise analysis failed to unveil a statistically robust rich club strucure for the left HC. We believe that this is due to an increased false positive rate. However, a “laterality” might be present in the HC with respect to rich club organization, a potentiality demanding further future elaboration. Network analysis in the macaque [53] and the human brain [70] revealed that the rich club connections are the most “costly”, i.e. span long distances, and mediate traffic between distant regions through a sequence of short-long-short range structural pathways. Studies in the human brain indicate that inter-regional functional interactions are modulated by connection distance and take place within specific frequency bandwidths [71], [72]. Additionally, macaque studies suggest a frequency-specific dialogue between two cortical regions that depends on the laminar origin and termination of the inter-regional connections [73]. Our comparative analysis can guide invasive techniques for the functional examination of the rich club regions of the MC. Such investigation is crucial for assessing if and how the aforementioned factors co-shape the functional dialogue within rich-club and between rich club and non-rich club regions and thus highlight the principles that shape the flow of information through this structural backbone. Additionally, such functional investigation might unlock the mechanisms underlying the proposed role of rich-club regions in multisensory integration [74]. Our comparative approach helps translating such functional findings to the human brain and develop hypothesis that could be tested with e.g. electrocorticography. In that way, future studies could assess if “homologous rich club” regions exhibit comparable and/or unique functional properties in the two species.

Lesions involving rich-club regions deteriorate the efficiency of the whole brain network and consequently can affect multiple cognitive domains as well as functional aspects like synchronization of functional networks [22]. The presence of a rich club structure involving highly overlapping regions in both MC and HC suggest that the macaque brain might be used as a model for e.g. studying the effects of lesions involving “homologous rich club” regions. However, certain common rich club regions, for instance CCp, lack significant interspecies connectivity similarity. Lesions in a brain region, apart from leading to the expected effects in regions directly connected to it, also lead to global effects through indirect connections [75]. Thus, if the wiring of the same lesioned regions differs, the lesion can lead to different global effects and consequently possibly different behavioural effects. The above conjointly, suggest that while lesioning common rich club regions will have detrimental global effects in both species, the nature and severity of such effects might depend on the degree of preservation of the connectivity of the involved regions.

### Factors responsible for connectivity discrepancies between the species

Both genetic and environmental factors underlie system-level changes, including connectivity, of the cortex of mammals [12]. For instance, functional connectivity differences observed between the inferior parietal lobule and anterior prefrontal cortex of macaques and humans can be the result of different foraging styles of the two species, dictated by different ecological factors which entail different challenges in decision making [65]. Our results revealed statistically significant connectivity similarities between humans and macaques while absence thereof might suggest a rewiring also caused by the aforementioned factors. In addition, inaccuracies of the methods used and data incompleteness might also give rise to connectivity discrepancies (see Limitations and future directions). Thus, discrepancies might be attributed to “true” differences, methodological limitations and a mixture thereof.

### Does a statistically significant connectivity similarity necessarily entail functional similarity?

Both empirical and computational studies suggest that the connectivity of a region largely constrains its function [8], [9]. We have demonstrated the perseverance of the connectional patterns of certain assumed homologues in the two species in a quantitative way. Obviously humans and monkeys differ in certain cognitive functions e.g. language production. Hence, one intriguing question is the extent to which such a statistically significant perseverance of connectivity similarity is translated to similarity of function persevering evolution. Other factors apart from macroscale connectivity can shape the functional role of a region, e.g. laminar patterns of connections [76]. Consequently, it could be the case that a statistically significant macroscale connectivity similarity of a region is not sufficient to guarantee evolutionary preserved functional similarity. In an analogous way, it has been demonstrated that the presence of an evolutionary conserved network can be accompanied by functional divergence [77]. Hence, while a statistical perseverance of macroscale connectivity suggests functional similarity, such a prediction demands explicit quantification in future studies. The network based methods employed in the current study in conjunction with data-driven methods for detecting cross-species functional homologies [78] could be adopted in future studies for addressing the degree of convergence and divergence of connectional and functional similarity across the brain regions of the two species.

### Limitations and future directions

Certain limitations should be taken into account when interpreting the findings of our study. First, while the expansion model is used extensively for interspecies comparisons, evidence suggests the presence of interspecies functional correspondences not predicted by it [78], [79]. To perform interspecies comparisons without using the expansion model, dwMRI and/or resting–state fMRI data collected in both species in conjunction with sophisticated techniques like network alignment [80] can be used for an interspecies connectivity based region-to-region match. Moreover, connectivity based parcellation strategies can be adopted for parcellating the cortical mantle in a data-driven fashion [58], [61] without the need for an a priori defined parcellation scheme. This would allow addressing inter-species differences and similarities of connectional architecture at a more fine grained level going beyond the level of granularity currently adopted. However, performing connectivity based whole brain parcellation applicable to a comparative study remains challenging. Second, the MC was assembled through a meta-analysis of tracing studies, while the HC with the aid of dwMRI. Good correspondence exists between the structural connections as revealed by tracers and diffusion imaging [21], [81], [82], but some inconsistencies are also discernible [38]. Hence, we predict that the usage of dwMRI for assembling the MC will lead to largely comparable results. Moreover, different weighting schemes for assembling the HC could also be adopted in the future [22]. Third, tractography methods have several limitations, like the limited detailed controlateral connectivity and the relation of false-positives and false negatives and connection distance, with longer connection distances appearing more prone to false negatives [38]. Hence, connections between distant regions might be underrepresented and might lead to lack of interspecies connectivity similarity (for a discussion see [24], [83]. Future studies employing the same modality for the estimation of connectivity in the two species, e.g. resting-state fMRI, will complement the current results. Forth, we currently used binary instead of weighted connectomes for the main analysis since certain network metrics currently employed (e.g. HCS) involve cross-matrix operations and the weights obtained from the different methods for assembling the MC and HC are not comparable. This restricted us from using all the metrics for the hemisphere-wise weighted analysis. Fifth, we compared the macroscale connectivity of the two species [20]. Apart from similarities and changes occurring at this level, connectivity changes between the species can occur at a mesoscale, i.e. connectivity at the laminar level [14], [84]. Hence, a more complete understanding of interspecies differences requires quantitative comparative studies at multiple levels. Lastly, future incorporation of whole brain macroscale connectivity data from more primate species, e.g. apes, along with enrichment of existing connectivity databases and improvement of neuroinformatics tools [49], [85], will allow tracing the evolutionary trajectory of the primate brain in more detail. To this end, network metrics recently introduced for uncovering the structural backbone of the brain, such as core-periphery analysis [86], could also be used as an alternative to the metric, i.e. rich-club, currently employed.

### Conclusions

We examined at the whole brain level the macroscale inter-regional structural connections of macaques and humans. While many similarities of the macroscale connectivity of the two species were observed, certain discrepancies were also present. This approach, which can be termed “comparative connectomics”, offers closer interspecies comparisons and brings forth novel insights into the evolution of the connectional architecture of the primate brain. Thus, it constitutes a translational bridge, valuable for clinical, cognitive and systems neuroscience, between macaque and human research.

## Supporting Information

Figure S1 Scatterplots of centrality and clustering values for the MC and HC. A. BC B. EC and C. C values. Lines represent a least-square fit. Correlations between values obtained from the MC and HC were not significant (see Results).
(TIF)

Figure S2 Region-wise values obtained for A. HCS and B. HMIS. Black squares and black circles represent the values obtained from the unscrambled and scrambled MC and HC respectively. Boxplots represent the null values obtained from matched random networks and depict the median, 25% and 75% quantiles, and outliers of the null values.
(TIF)

Figure S3 Curves depicting the values for the RCC for a range of k values for both A. MC and B. HC. Green curves and red curves correspond to values obtained from the unscrambled and scrambled networks respectively. Blue curves correspond to values obtained from matched random networks. Note that both the green and red curves lie above the blue ones that represent the null values for the RCC.
(TIF)

Table S1 RM acronyms and full names of the regions constituting the whole brain parcellation used in the current study. Each region is classified as allocortical (Allo), isocortical (Iso) or subcortical (Sub). An assignment is also provided based on a 5 way division (F = frontal, T = temporal, P = parietal, O = occipital, L = limbic, I = insular).
(XLS)

Table S2 Weighted HMIS results of the hemisphere wise analysis for the RM regions. Regions exhibiting significant HMIS values (p<0.05 FDR corrected) are in bold. The p-values and z-scores of the aforementioned metrics are also depicted.
(XLS)

Table S3 Centrality and clustering metrics of the RM regions for whole brain binary network analysis. Metrics in bold indicate that the specific region exhibits a metric with values higher than the mean+1 std (for the BC and EC) and lower than the mean+1 std (for C). The mean and std is calculated across the values obtained for each metric for every RM region. Regions with more than two metrics in bold are highly central and less segregated.
(XLS)

Table S4 Centrality and clustering metrics of the RM regions for hemisphere-wise and weighted analysis. Metrics in bold indicate that the specific region exhibits a metric with values higher than the mean+1 std (for the BC and EC) and lower than the mean+1 std (for C). The mean and std is calculated across the values obtained for each metric for every RM region. Note that values for BC and C are normalized.
(XLS)

Table S5 List of regions constituting the rich club in macaque and human for whole brain binary network analysis. The set of regions corresponds to the level k for which the maximum normalized RCC was observed (k = 56 for macaque and k = 55 for human) (see Fig. 4). Regions in bold denote rich club regions common in both species. Coordinates for the macaque correspond to the F99 space and for the human to MNI space. The suffixes R and L denote the right and left hemisphere.
(XLS)

Table S6 List of regions constituting the rich club for the hemisphere-wise and weighted analysis for the macaque (left hemisphere). The set of regions corresponds to the level k for which the maximum normalized RCC was observed (k = 32). Coordinates correspond to the F99 space.
(XLS)

Table S7 List of regions constituting the rich club a for the hemisphere-wise and weighted analysis for the macaque and human (right hemisphere). The set of regions corresponds to the level k for which the maximum normalized RCC was observed (k = 33 for macaque and k = 31 for human). Regions in bold denote rich club regions common in both species. Coordinates for the macaque correspond to the F99 space and for the human to MNI space.
(XLS)

Table S8 HCS and HMIS values for the BB 47 regions for whole brain binary network analysis. Regions exhibiting significant HCS and HMIS values (p<0.05 Bonferroni corrected) are in bold. The p-values and z-scores of the aforementioned metrics are also depicted.
(XLS)

Table S9 List of regions constituting the rich club in macaque and human based on the BB 47 parcellation scheme for whole brain binary network analysis. The set of regions corresponds to the level k for which the maximum normalized RCC was observed (not shown). Regions in bold denote rich club regions common in both species. Coordinates for the macaque correspond to the F99 space and for the human to MNI space. The suffixes R and L denote the right and left hemisphere.
(XLS)

Software S1 MATLAB code for calculating the HCS index.
(DOC)
