# Supplementary figures and images for: Comparative Analysis of the Macroscale Structural Connectivity in the Macaque and Human Brain
Source: PLoS Comput Biol. 2014 Mar 27;10(3):e1003529. doi: 10.1371/journal.pcbi.1003529 (PMC3967942; doi:10.1371/journal.pcbi.1003529)

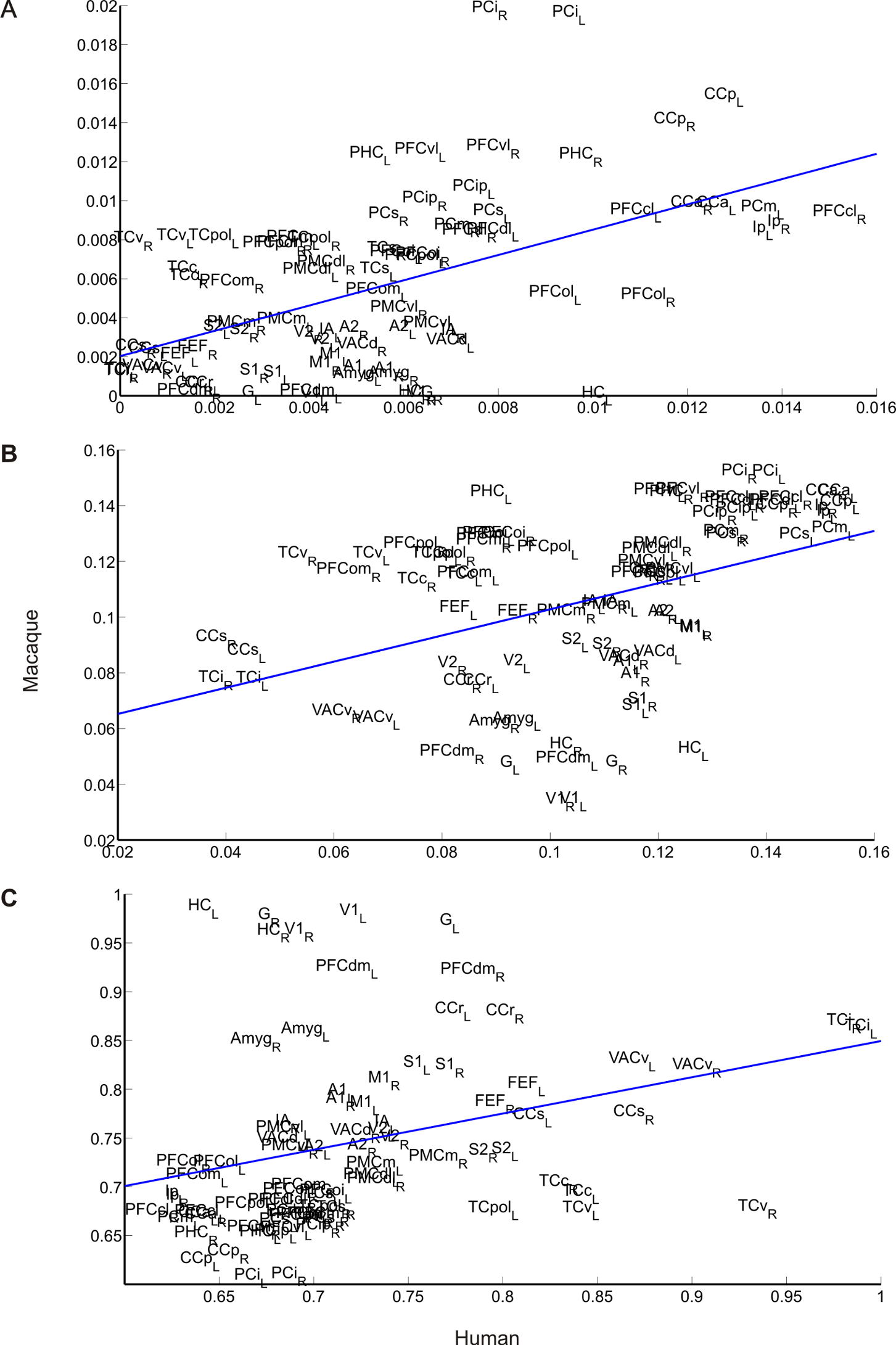

Supplement: Figure S1 — Scatterplots of centrality and clustering values for the MC and HC. A. BC B. EC and C. C values. Lines represent a least-square fit. Correlations between values obtained from the MC and HC were not significant (see Results). (TIF) [file pcbi.1003529.s001.tif]

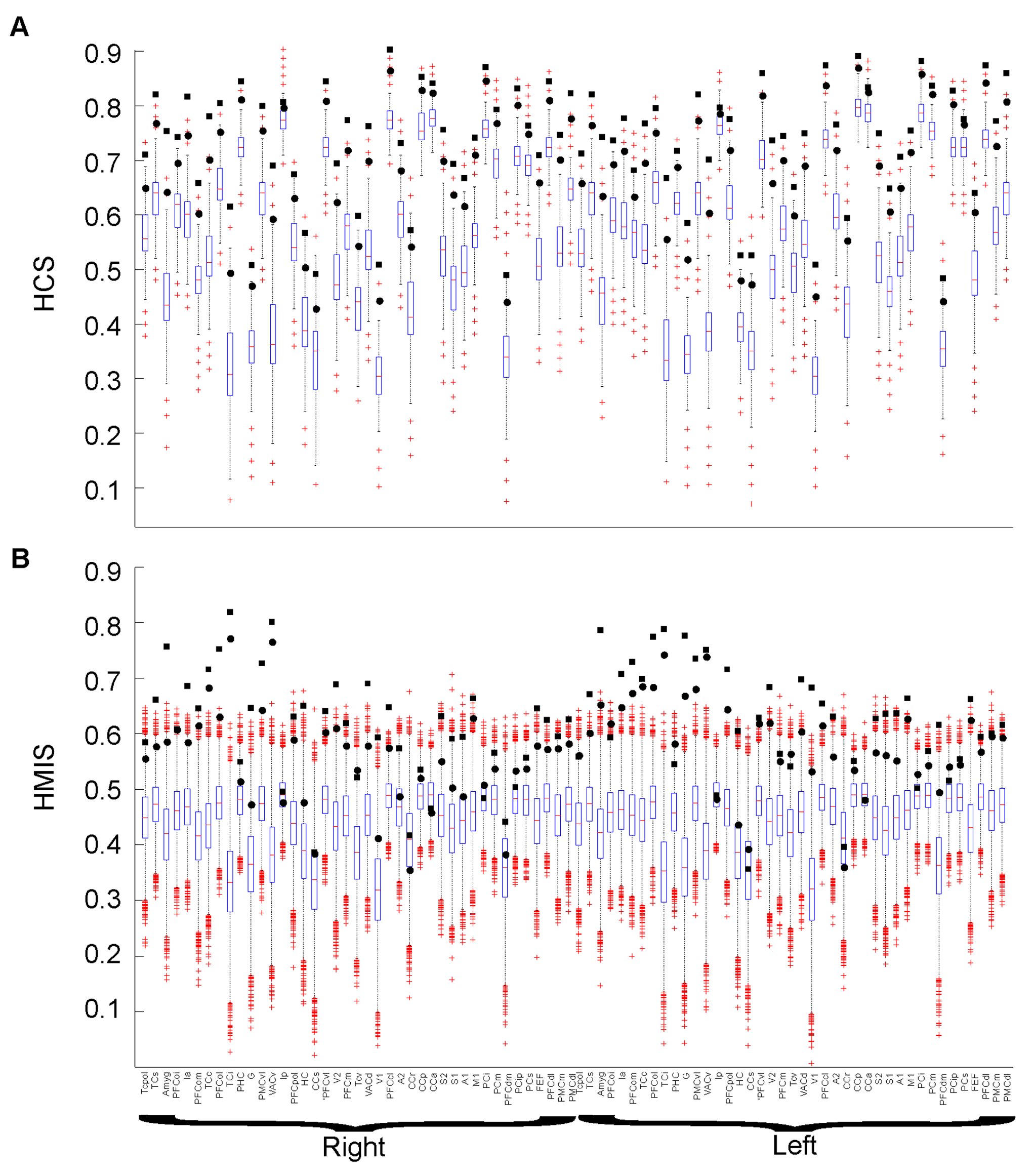

Supplement: Figure S2 — Region-wise values obtained for A. HCS and B. HMIS. Black squares and black circles represent the values obtained from the unscrambled and scrambled MC and HC respectively. Boxplots represent the null values obtained from matched random networks and depict the median, 25% and 75% quantiles, and outliers of the null values. (TIF) [file pcbi.1003529.s002.tif]

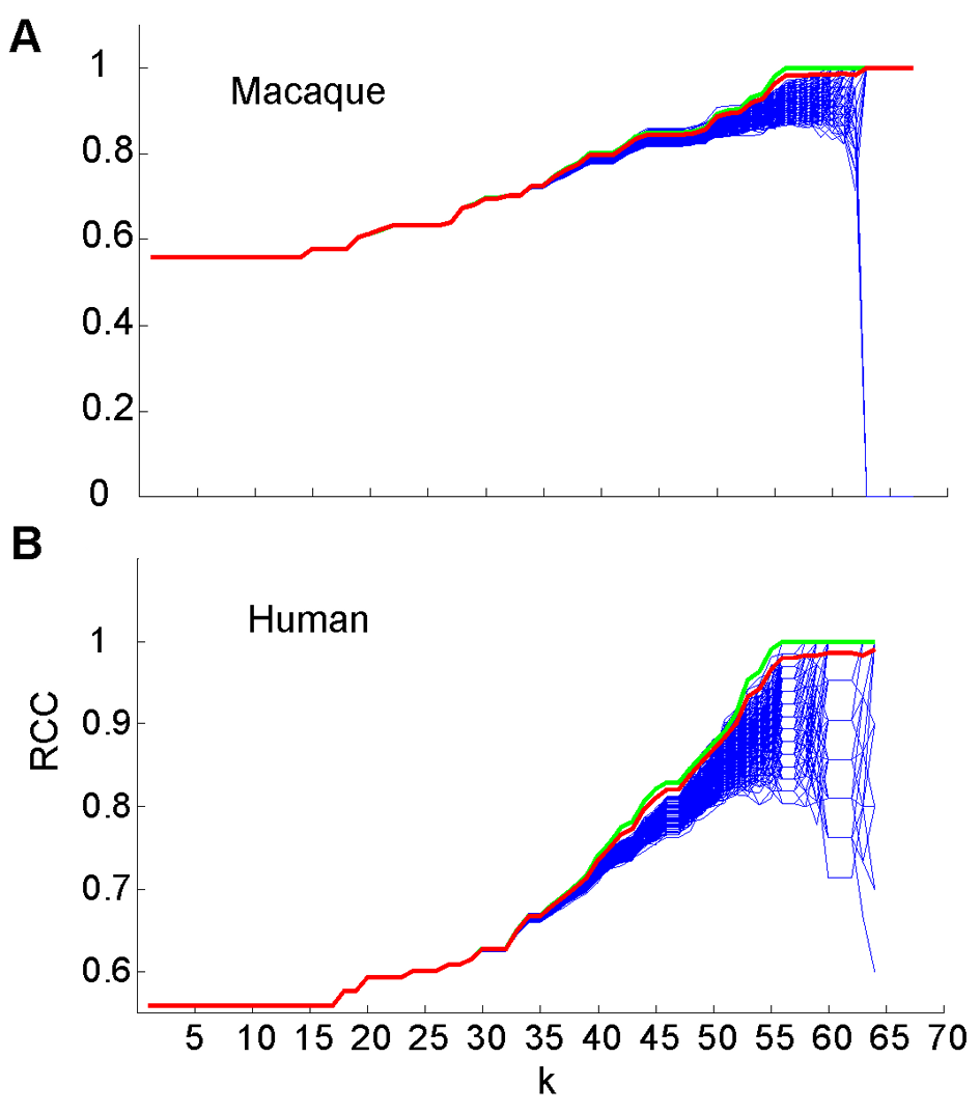

Supplement: Figure S3 — Curves depicting the values for the RCC for a range of k values for both A. MC and B. HC. Green curves and red curves correspond to values obtained from the unscrambled and scrambled networks respectively. Blue curves correspond to values obtained from matched random networks. Note that both the green and red curves lie above the blue ones that represent the null values for the RCC. (TIF) [file pcbi.1003529.s003.tif]
