# Supplementary material for: Comparative Analysis of the Macroscale Structural Connectivity in the Macaque and Human Brain
Source: PLoS Comput Biol. 2014 Mar 27;10(3):e1003529. doi: 10.1371/journal.pcbi.1003529 (PMC3967942; doi:10.1371/journal.pcbi.1003529)
Supplement: Software S1 — MATLAB code for calculating the HCS index. (DOC) [file pcbi.1003529.s013.doc]

%The script compares the connectivity patterns of all nodes in A and B.

%This is a cross-matrix variation of the "classic" matching index and thus

%follows the same rationale.

%

%Input:

%A,B: NxN binary symmetric connectivity (adjacency) matrices.

%mode: 1=Compute region-to-region cross-matrix MI

% 2=Compute region to all region cross matrix MI

%computationmode: 1=calculate MI as intersection/overlap of connections

% 2=calculate MI as in BCT: common connections/total

% number of connections*2 (so it scales to 1 as max value)

%Output:

%CMI A vector of cross matrix MIs (if mode=1)

% A NxN matrix of cross matrix MIs (if mode=2)

%--------------------------------------------------------------------------

function CMMI=CrossMatrixMI(A,B,mode,computationmode)

if(mode==1)

CMMI=zeros(length(A),1);

for i=1:length(A)

if(computationmode==1)

CMMI(i,1)=length(find((A(i,:)+B(i,:))==2))/length(find((A(i,:)+B(i,:))~=0));

else

CMMI(i,1)=2*(length(find((A(i,:)+B(i,:))==2))/(sum(A(i,:))+sum(B(i,:))));

end

end

else

CMMI=zeros(length(A),length(A));

for i=1:length(A)

for j=1:length(B)

A1=A(i,:);

B1=B(j,:);

A1([i j])=0;%Wipe out direct connections between regions for which we compute the cross-matrix MI.

B1([i j])=0;

if(computationmode==1)

CMMI(i,j)=length(find((A1+B1)==2))/length(find((A1+B1)~=0));

else

CMMI(i,j)=2*(length(find((A1+B1)==2))/(sum(A1)+sum(B1)));

end

end

end

end

return;
